# Pro-apoptotic liposomes-nanobubble conjugate synergistic with paclitaxel: a platform for ultrasound responsive image-guided drug delivery

**DOI:** 10.1038/s41598-018-21084-8

**Published:** 2018-02-08

**Authors:** Rajeet Chandan, Rinti Banerjee

**Affiliations:** Department of Biosciences and Bioengineering, Indian Institute of Technology Bombay, Mumbai, India

## Abstract

Recently, liposomes-microbubble conjugates have emerged as a promising ultrasound (US)-responsive platform for cancer therapeutics. However, these are limited by their size in terms of tumor penetration. Additionally, there have been no attempts to enhance the smartness of such conjugates which have been used only as passive carriers. The present study explores submicron sized (756 ± 180.0 nm), US-responsive, phosphatidylserine (PS)-based paclitaxel-liposomes-nanobubble conjugates (PSPLBC) with an additional pro-apoptotic effect towards enhanced anti-cancer efficacy and image-guidance. The developed PSPLBC underwent cavitation in response to US-trigger, exhibiting *in vitro* pulsatile release with a 10-fold increase in cellular internalization as compared to control. The PS-containing formulations were found to be pro-apoptotic and exhibited strong synergism between PS and paclitaxel (Combination Index, CI < 0.1). This resulted in significantly high anti-tumor efficacy both *in vitro* and *in vivo* conditions (98.3 ± 0.8% tumor growth inhibition, TGI). Significant reduction in tumor proliferation index and MVD, as well as significant increase in apoptosis, were observed for the treated tumor sections. Further, the intravenous (i.v.) administration of PSPLBC enhanced the tumor US-contrast by 2-fold as compared to SonoVue. These results, show the potential of PSPLBC as a promising non-invasive, pro-apoptotic, smart DDS for US-responsive, image-guided cancer therapeutics.

## Introduction

The field of precision medicine has progressively benefited from the developments in nanotechnology. The incorporation of image-guidance capability to the external trigger-responsive DDSs have further pushed the envelope of the precision medicine in overcoming the drawbacks, *viz*. toxicity and nonspecificity, of the conventional cancer chemotherapy. Such systems allow monitoring of particles’ accumulation followed by on-demand triggered release of the chemotherapeutic(s) in the desired tissue^1,2^. This enhances the efficacy of the therapy as well as reduces the systemic toxicity associated with the free drug. Additionally, it also allows continuous monitoring of the disease response throughout the treatment regime. These promising features have gained considerable attention, leading to the development of various kinds of image-guided DDSs. This primarily includes; MR-guided magnetic field-responsive platforms, optically-guided light-responsive, US-responsive, electrothermally-responsive and X-ray-responsive platforms, and the like^2^. Since, US is safe, portable, affordable, patient compliant and have a wide reach in the healthcare facilities; the US-responsive platforms are of choice.

The US-responsive platforms emerged from the serendipitous discovery of microbubbles (MBs) by Gramiak and Shaw in 1968^3^. Since then MBs have covered long and successful journey to clinics as ultrasound contrast agents (UCAs). The MBs show both linear and non-linear acoustic backscattering of the incident ultrasound waves, generating strong echo signals, thereby enhancing the contrast substantially. Apart from backscattering, MBs also exhibit other acoustic phenomena like streaming, cavitation, radiation, and fragmentation depending upon the insonating US-wave as well as the physico-mechanical properties of MBs (size and shell elasticity)^4,5^. Out of these phenomena, cavitation is associated with relatively high mechanical and thermal energy and hence, is of considerable interest for drug delivery applications. Various studies have shown temporary deformation of the cell membranes by oscillating MBs^6,7^. These deformations provide a passive, concentration-dependent route of material exchange between extracellular and intracellular space, a property useful for drug/gene delivery. Additionally, the cavitational forces have also been reported to increase the permeability of physiological barriers such as blood-brain barrier and endothelium^8^. These features make MBs an attractive trigger-responsive system for drug/gene delivery. In order to exploit these features, several studies have explored the antitumor efficacy of the physical mixture of free drug and MBs. These studies have shown significant enhancement in cellular permeability leading to a considerable increase in cellular uptake of the drug *in vitro*^9,10^. However, these formulations have exhibited limited success in animal models^11^. It is primarily due to the fact that, the cavitation is a short-range force and hence, the close physical proximity of the MBs with cells and drug-carrier is a pre-requisite^6,9^. To overcome this shortcoming, drug-loaded MBs were explored, which were seen to be limited by their poor payload capacity and stability^10,12^. In order to improve their therapeutic performances, alternative approaches like on-site bubble generation and liposomes-MB conjugates have been explored^13–22^. The liposomes-MB conjugates have demonstrated significantly high drug loading capacity as well as improved antitumor efficacy *in vivo*^14,15^. However, the therapeutic efficacy of such conjugates could be further improved by reducing their size from a few micrometers to submicrometer as suggested by Cai *et al*.^23^. They reported that the nano-sized bubbles (565.2 ± 201.5 nm) had improved stability and enhanced passive tumor targeting as compared to micron size microbubbles (SonoVue) *in vivo*^23^. These nanobubbles also exhibited substantially high contrast-enhancement for an extended duration as compared to SonoVue. These findings suggested the advantages of submicron-sized constructs towards efficient tumor targeting as well as enhanced echogenicity for image-guided therapeutic applications over MB-based constructs.

Further, cancer is a complex disease with multiple genetic and phenotypic aberrations. Therefore, the management of the disease is often ineffective with the use of a single drug and requires a combinational drug approach. The combination of two drugs or a drug with an apoptotic agent has been shown to significantly improve the anti-tumor efficacy^24,25^. However, to the best of our knowledge, no attempts have been made to improve the smartness of drug-carrier-MB conjugates by incorporating a pro-apoptotic biomaterial in the drug carrier. Therefore, we have developed a smart pro-apoptotic paclitaxel-loaded liposome and conjugated it with nanobubbles for the combination therapy. In this study, we have evaluated the *in vivo* antitumor efficacy as well as US-contrast enhancement property of this conjugate for image-guided cancer therapeutics in a clinical setting.

## Results and Discussion

### Physicochemical characterization

**Figure 1:**
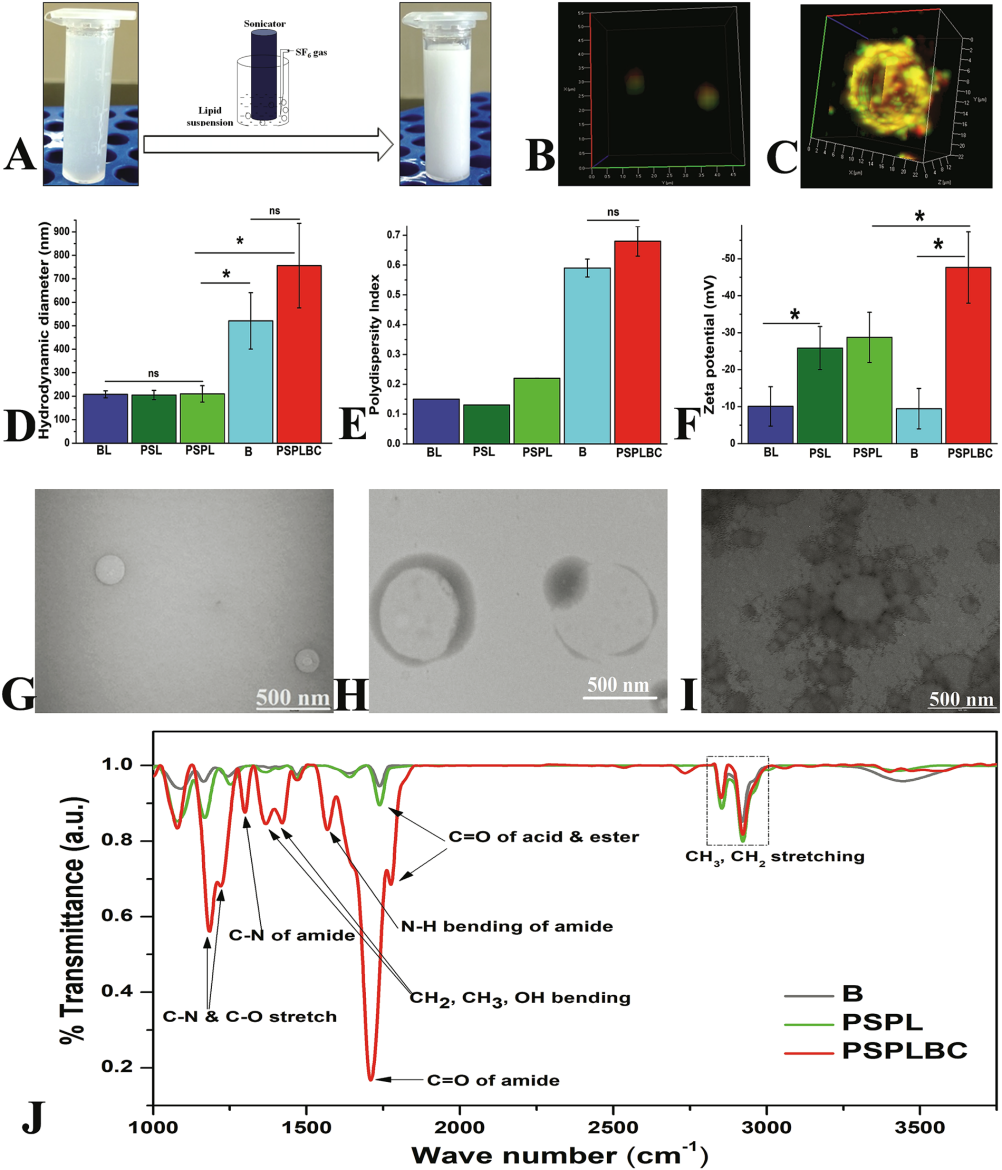
Physicochemical characterization studies. (**A**) Photograph showing nanobubble formation. The 3D merged confocal image showing colocalization of green (liposome) and red (nanobubble) signal; (**B**) submicron sized PSPLBC (scale bar 0.5 µm), (**C**) micron sized PSPLBC (scale bar 2 µm). (**D**) Hydrodynamic diameter, (**E**) polydispersity index, and (**F**) zeta potential of BL, PSL, PSPL, B and PSPLBC (*p ≤ 0.05 and ns = no significance). (**G**) PSPL, (**H**) nanobubble and (**I**) PSPLBC shows the TEM-images. (**J**) FTIR spectra of B, PSPL, and PSPLBC recorded in percent transmittance-mode using KBr pellet method.

The formation of nanobubbles was marked by the transformation of translucent lipid suspension into a milky suspension upon sonication with continuous gas purging, as shown in Fig. 1A. Next, the liposomes were prepared and conjugated to the nanobubbles *via*. covalent amide linkage using EDC/NHS chemistry between the COOH groups of the DOPS moiety present on the liposomes and NH_2_ groups of the DSPE moiety present on the nanobubbles. The detail mechanism of the standard EDC/NHS chemistry is shown in Figure S1. The degree of conjugation was optimized by varying the liposomes:nanobubble ratios used for conjugation reaction, followed by the estimation of the percent conjugate population using FACS (double positives). The scatter plots showed a gradual increase in the conjugate population from 47.8% to 91.4% with decreasing liposomes (PSPL):nanobubble (B) ratio from 9.9:0.1 to 9.0:1.0 as shown in Figure S2. The further decrease in the liposomes:nanobubble ratio led to the formation of larger conjugates. Hence, 9.0:1.0 ratio of PSPL:B was considered optimum and used for the preparation of PSPLBC. Also, the dual dye-loaded PSPLBCs were imaged using CLSM as a visual proof of the conjugation. Figure 1B shows co-localization of both red (nanobubble) and green (liposomes) signals suggesting successful conjugation and formation of uniformly distributed submicron-sized (~800 nm) PSPLBC. However, due to the resolution limit of the optical microscope, it was difficult to visualize the arrangement of the liposomes on the nanobubble surface. Therefore, micron-sized (~16 µm) PSPLBCs were prepared and visualized in a similar way. Figure 1C shows the merged 3D-image created from the z-stack of the micron-sized PSPLBC (the individual fluorophore channels are shown in Figure S3). The 3D-image clearly showed the co-localization of green signals (calcein loaded liposomes) on the surface of a hollow red sphere (Nile-red labeled bubble), validating the conjugation of liposomes on the surface of nanobubbles/bubbles, as illustrated in Figure S4.

Followed by the successful synthesis, the particles were characterized for their size, zeta potential, and morphology. The hydrodynamic diameters were measured to be 521 ± 120 nm for nanobubbles, 756 ± 180 nm for PSPLBC and 208 ± 15 nm, 205 ± 20 nm and 210 ± 35 nm for liposomal formulations *viz*. BL, PSL, and PSPL respectively (Fig. 1D). The polydispersity indices were recorded to be 0.59 ± 0.03 for nanobubbles, 0.68 ± 0.05 for PSPLBC and 0.15 ± 0.001, 0.13 ± 0.001, and 0.22 ± 0.002 for liposomal formulations *viz*. BL, PSL, and PSPL respectively (Fig. 1E). Polydispersity index (PDI) is the measure of the heterogeneity in the particle size distribution. Colloidal suspensions with low particle size heterogeneity have low PDI value and vice-versa. The PDI values show that the liposomal formulations have narrow size distribution (monodispersed) as compared to the nanobubbles and the conjugates. Though, the nanobubbles and the conjugates have slightly higher PDI values, it is significantly lower than the 1.05 ± 0.24 PDI recorded for the SonoVue (commercial microbubble formulation) using DLS. Also, the size distribution of the nanobubbles and the conjugates is significantly narrower as compared to that of the commercial microbubble formulation (SonoVue, 2–8 µm)^26^. Zeta potential is a critical parameter that determines the colloidal stability. A higher magnitude of zeta potential lead to electrostatic repulsion between the particles owing to improved dispersibility, conferring stability to the suspension. The zeta potentials were found to be −47.6 ± 9.7 mV for PSPLBC, −9.5 ± 5.5 mV for nanobubbles and −10.1 ± 5.3 mV, −25.8 ± 5.8 mV and −28.7 ± 6.8 mV for liposomal formulations *viz*. BL, PSL, and PSPL respectively (Fig. 1F). The BL were composed of neutral lipids, whereas, the PSL, PSPL liposomal formulations contained DOPS (an anionic lipid). Hence, the zeta potential of the BL was low as compared to PSL and PSPL. Like BL the nanobubbles were also composed of neutral lipids and hence, bears very low zeta potential of −9.5 ± 5.5 mV. However, this value improved significantly (−47.6 ± 9.7 mV) due to the tethering of the negatively charged liposomes (PSPL, −28.7 ± 6.8 mV) to the nanobubbles to form PSPLBC (final formulation). The increased zeta potential indicates successful conjugation as well as confers colloidal stability to the PSPLBC. Further, the TEM images revealed uniformly distributed spherical particles for liposome and nanobubble samples (Fig. 1G and H). For PSPLBC, pendant-like structures with the liposomes attached on the surface of the nanobubbles were observed (Fig. 1I). Further, to investigate the formation of amide bond, the FTIR-spectra were recorded. It showed an appreciable shift in the carbonyl stretching peak from 1776 cm^−1^ to 1710 cm^−1^ signifying the transition of the acid carbonyl group to amide carbonyl group. Also, two new peaks emerge at 1568 cm^−1^ and 1298 cm^−1^ which correspond to N-H bending and C-N bending of the amide-bond respectively (Fig. 1J). These peaks confirmed the successful participation of the -COOH groups of the DOPS moiety present on the liposomes and the -NH_2_ groups of the DSPE moiety present on the nanobubbles, towards the formation of amide bonds. These data *viz*. confocal images, hydrodynamic diameter, PDI, zeta potential, TEM images and FTIR spectra, shows the structural integrity of the PSPLBC as well as proves the covalent conjugation of the nanobubbles and the liposomes to form PSPLBC.

The lyophilized PSPLBC powder quickly and completely re-dispersed into milky suspension, which separated into lower clear solution and upper thick layer of PSPLBC particles, as shown in Figure S5. No significant size change was observed for the reconstituted lyophilized particles over months. The stability of the PSPLBC formulation in suspension was also accessed over time by measuring the particle size at various time points. The formulation was found to be stable for a week at 4 °C storage condition. This is significantly higher than 6 hours of stability of SonoVue after reconstitution^26^. Therefore, PSPLBC is a stable powder formulation that can be reconstituted at bed-side.

### Drug Loading and triggered release

Lipid bilayer of the liposomes provides extended space for hydrophobic interactions resulting in high encapsulation efficiency of the hydrophobic drugs. Therefore, liposomes were chosen as the drug-carrier for this study. The developed liposomes (PSPL) exhibited 86.4 ± 2.8% paclitaxel entrapment efficiency, which translated into 27.3 ± 0.7% (w/w) drug loading capacity of the PSPLBC. Such an increase in drug loading capacity can be attributed in parts to the high encapsulation efficiency of PSPL as well as large effective surface area provided by the nanobubbles for tethering of the PSPL.

Next, the triggered release was studied using pulsed US-triggers of intensity 2 W/cm^2^ (PNP = 0.17 MPa) at 1 MHz frequency for 60 seconds, translating to 11.9 Joules of US-energy per ml of sample was applied at an interval of 60 minutes.

**Figure 2:**
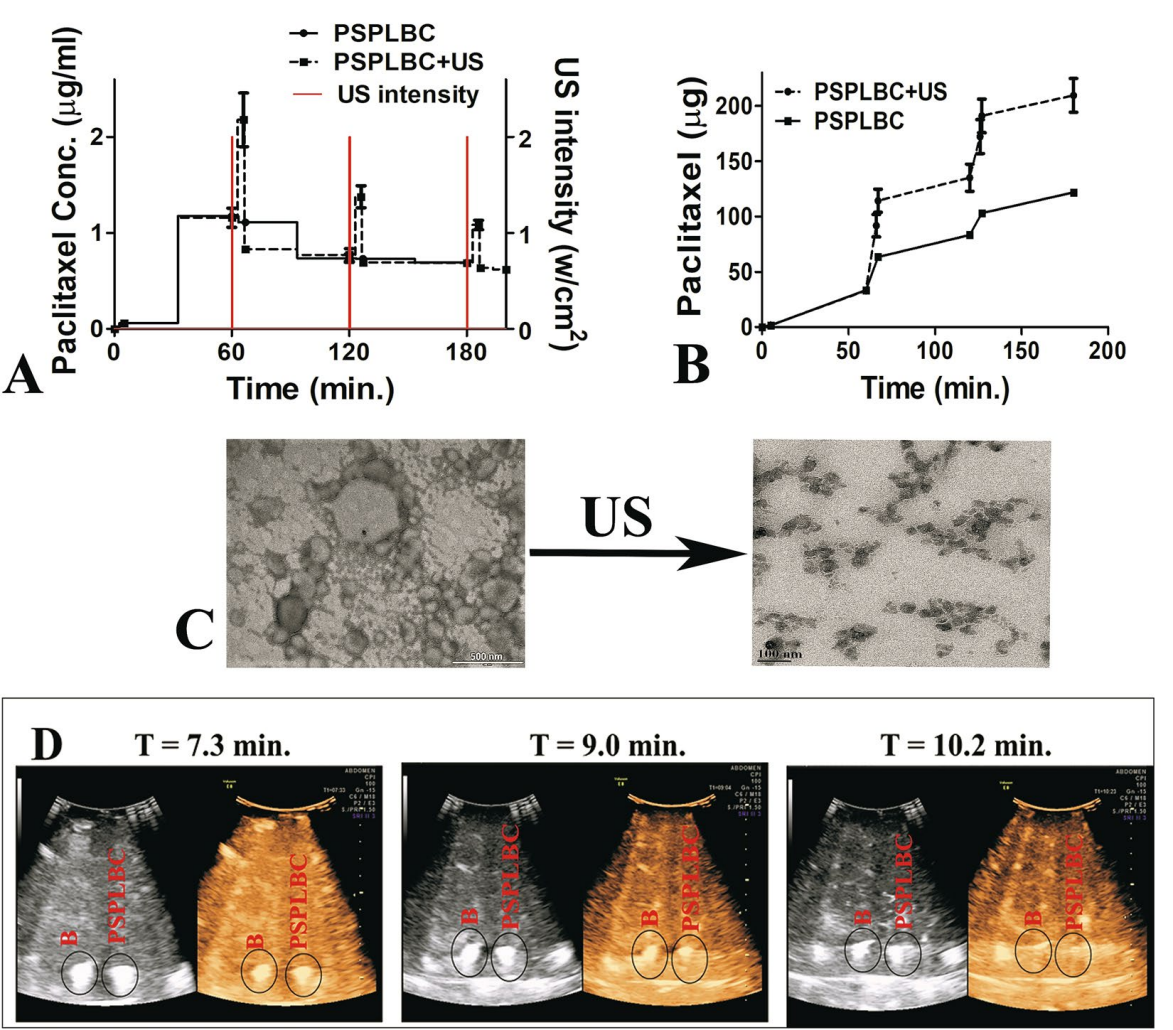
*In vitro* triggered release study. (**A**) Shows the US-trigger intensity profile and the concentration of released paclitaxel in sink solution; (**B**) Shows the cumulative release plot, for both with US-trigger. (**C**) Shows the TEM images of PSPLBC before and after US-trigger. The observed lipid fragments confirmed the complete destruction of intact PSPLBC into lipid fragments upon US exposure. (**D**) Ultrasonography of B and PSPLBC in both B-mode (grey) and contrast mode (Solaris) using 5–7 MHz probe.

The application of US-trigger resulted in a non-linear release profile. The trigger led to an immediate increase in the paclitaxel concentration giving rise to sharp paclitaxel concentration peaks, which overlapped with the trigger intensity peaks on the temporal frame (Fig. 2A). In the absence of trigger i.e., without trigger or in between the triggers, the PSPLBC exhibited slow diffusional release profile. This shows the US-responsiveness of the PSPLBC as well as their on-demand pulsatile release profile. The cumulative release plot exhibited sustained diffusional release accounting for 24.5 ± 2.5% of paclitaxel release without trigger whereas, a pulsatile 74.8 ± 5.7% with trigger (Fig. 2B). It is comparable to 74.39% triggered paclitaxel release reported by Yan *et al*. using similar microbubble-liposome construct^14^. Upon application of the US-trigger, the nanobubble core of the conjugate (PSPLBC) undergo vigorous compression and expansion cycles and finally undergo cavitation. The cavitation is associated with physical forces that can compromise/disrupt the liposomal membrane, releasing the drug molecules. To investigate this, the TEM analysis of the PSPLBC before and after US-treatment was done (Fig. 2C). The TEM images show complete structural disintegration of the nanobubble core as well as the tethered liposomes into lipid fragments. It suggests the complete loss of liposomal bilayer integrity due to the cavitation, as the mechanism for the observed on-demand trigger-responsive pulsatile release; a desired property for the precision medicine.

### *In vitro* echogenicity

After establishing the US-responsiveness, the *in vitro* contrast enhancement potential of the developed PSPLBC was evaluated using a clinical US scanner with a 5–7 MHz phased array convex probe. The ultrasonography experiments were performed in both B-mode and CEUS-mode with a sample depth of ~10 cm. The sonography exhibited visibly similar bright contrast for both nanobubbles and PSPLBC (Fig. 2D). Since the contrast generated in CEUS-mode selectively utilizes secondary/tertiary harmonics, (a characteristic feature of bubbles) the observed contrast confirmed the integrity of nanobubble core as well as ruled out any significant compromise in echogenic property of PSPLBC due to tethering of liposomes. Further, the time-lapsed ultrasonography showed a time-dependent gradual decrease in the contrast intensity, with the complete dissipation of the contrast in 10.2 minutes, for both B and PSPLBC (Fig. 2D). This enhanced contrast duration is desired, as it increases the time window for investigation as well for focusing of the US-beam for the therapy. These results, hence, confirmed the image-guiding potential of the PSPLBC using clinical US-scanner.

### *In vitro* studies

Reports suggest a direct detrimental effect of cavitation on cellular membranes and thus, the cell viability^27,28^. Therefore, the acoustic power of US-trigger was optimized to inhibit/minimize any direct detrimental effect of US-mediated cavitation on cell viability; confounding the evaluation of the effect of the formulation. A gradual decrease in cell viability with increasing trigger intensity was observed (Figure S6), suggesting detrimental effects at intensities above 2 W/cm^2^. The cells treated with an intensity of 2 W/cm^2^, 50% DC, 15 s (i.e. 12 Joules/ml of US-energy) exhibited more than 95% cell viability. Hence, US-trigger of the intensity of 2 W/cm^2^ at 50% DC for 15 seconds was considered safe and used for all the *in vitro* studies.

### Cellular internalization and its mechanism

**Figure 3:**
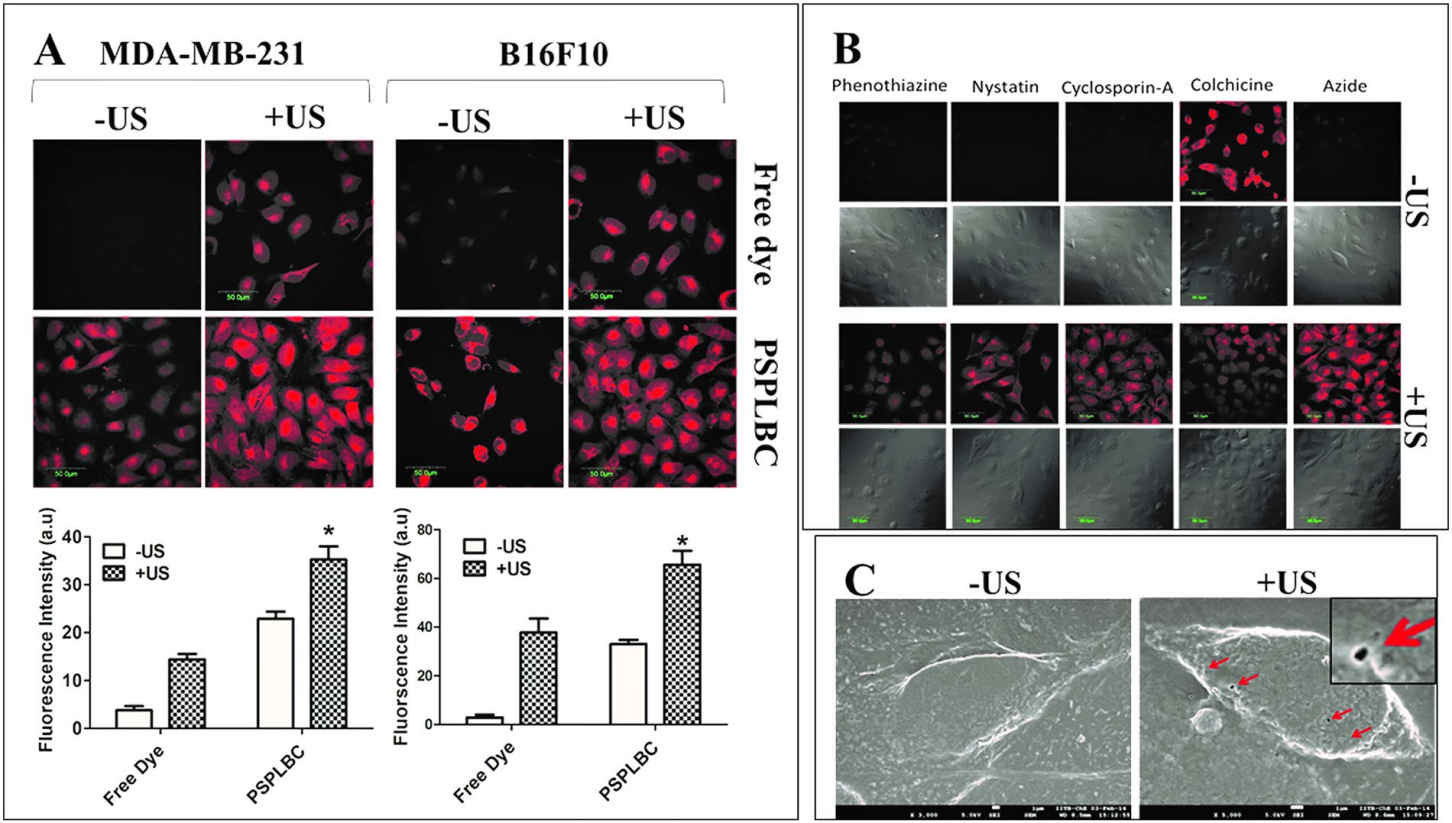
Cellular internalization study. (**A**) CLSM images of MDA-MB-231 and B16F10 cells treated with free rhodamine-6G dye, free dye+US, rhodamine-6G loaded PSPLBC, and rhodamine-6G loaded PSPLBC+US. The fluorescence intensity was quantified from three random CLSM images. (*p ≤ 0.05). (**B**) Mechanism of PSPLBC cellular internalization studied using cells pre-treated with various metabolic & endocytic inhibitors. CLSM images show the uptake of PSPLBC by pretreated cells, both with and without application of trigger. (**C**) Cryo-FEG-SEM images of the cell without US-treatment and immediately after PSPLBC+US treatment. The red arrows show the sonopores on the cell surface, the inset shows the zoom-in picture of a sonopore, (scale bar = 1 µm).

Cellular internalization was studied and quantified by estimating the fluorescence intensities of the respective confocal images. Weak fluorescence signals were observed for the free rhodamine-6G dye treated cells whereas, a significant increase in signal intensity was detected for the free-dye + US treated cells. A similar trend was observed for the rhodamine-6G loaded PSPLBC treated cells *i.e*. significant increase in fluorescence signal for the PSPLBC + US group as compared to the PSPLBC group. Among the free dye and PSPLBC groups, the PSPLBC treated cells exhibited substantially high fluorescence signal. Both the MDA-MB-231 and B16F10 cells showed a similar trend (Fig. 3A). Next, the Z-scans showed maximum intensity at the focal plane, confirming an appreciable intracellular accumulation of the particles (Figure S7). Further, the quantification of fluorescence intensity for the PSPLBC treated cells revealed a 6.0-fold increase for MDA-MB-231 and 11.5-fold increase for B16F10, as compared to the free dye treated cells. Subsequently, with the application of trigger, the fluorescence intensity of the PSPLBC + US treated cells increased 1.5-fold in MDA-MB-231 and 1.9-fold increase in B16F10 as compared to the PSPLBC treated cells (Fig. 3A). This significant enhancement in the cellular internalization of PSPLBC as compared to free-dye can be attributed to its biomimetic, fusogenic lipids, which could induce receptor-mediated endocytosis. Additionally, the application of US was seen to further enhance the cellular internalization significantly in each group. Therefore, to understand the role of constituent lipids and US-trigger in cellular internalization process, the mechanism of internalization was studied using various metabolic and endocytic blockers. Sodium azide was used as a metabolic blocker which blocks the synthesis of ATP. A weak fluorescence signal for sodium azide treated cells suggested the dependence of internalization on ATP (*i.e*. active process). To further investigate the pathway of endocytosis, various endocytic blockers *viz*. phenothiazine, nystatin, cyclosporine-A & colchicine which block the clathrin-dependent, caveolae-dependent, calcineurin-dependent, & microtubule-mediated endocytic pathways respectively, were used. The cells pretreated with the endocytic blockers exhibited weak fluorescence signals except for colchicine-treated cells (Fig. 3B). It suggested that the internalization of PSPLBC (no trigger) follow multiple endocytic pathways *viz*. clatherine, caveolae, & calcineurin-dependent pathways but is independent of microtubule-mediated endocytosis. On the other hand, the addition of US-trigger to the PSPLBC treated cells exhibited bright fluorescence signals for all the groups. It implies that the cellular internalization of PSPLBC + US treated cells is a passive process and is independent of the aforementioned endocytic pathways. As the sonopores provide non-selective, passive channels for cellular transport across the membrane. These intriguing results could be explained by the formation of sonopores^6,7,9,29^.

Therefore, in our pursuit to further understand the mechanism of cellular internalization of PSPLBC + US treated cells, the cellular membrane morphology of PSPLBC + US treated cells were examined by cryo-SEM. Figure 3C shows the appearance of pore-like structures of 100–400 nm size (marked with arrows) on the surface of the PSPLBC + US treated cells, whereas, no such structures were seen on the untreated cells. Both the confocal data (Fig. 3A) and the cryo-SEM data (Fig. 3C) indicates the formation of sonopores. These results also indicated the potential of PSPLBC to evade the endosomal route and directly deliver its cargo to the cytoplasm *via*. sonopores, an attractive route to bypass the endosomal pathway for pH labile drugs.

### *In Vitro* Cytotoxicity study

**Figure 4:**
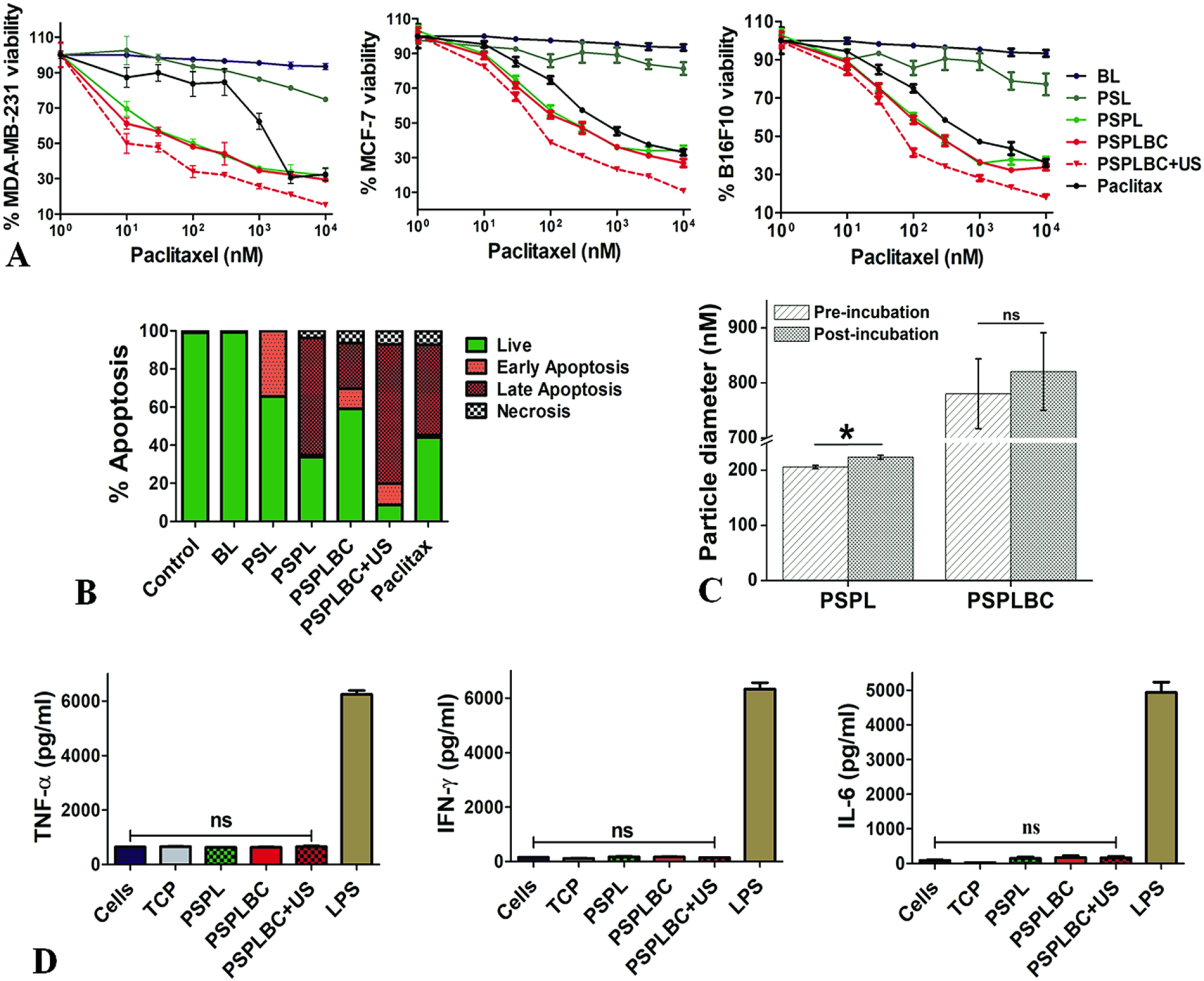
*In vitro* studies. (**A**) Shows the drug dose-response plots for MDA-MB-231, MCF-7, and B16F10 cells. (**B**) Shows stacked histogram for the percentage of the cellular population in different apoptotic phases after treatment with the respective formulation. (**C**) Hydrodynamic diameter of the PSPL and PSPLBC, pre- and post-incubation with FBS for 02 hours. (**D**) Shows the levels of secreted TNF-α, IFN-γ and IL-6 cytokines by the PSPLBC treated RAW cells (*p ≤ 0.05, ns = not significant).

The cytotoxic potential of BL, PSL, PSPL, PSPLBC, PSPLBC + US, and Paclitax was evaluated using MDA-MB-231, MCF-7, and B16F10 cells. Irrespective of the cell-line, no cytotoxicity was observed for the BL treated cells whereas, dose-dependent cytotoxicity was observed for rest of the formulations including PSL (Fig. 4A). The PSL contains only PS as active moiety (no drug) and hence, exhibited only a limited cytotoxicity which can be attributed to PS-induced apoptosis, as reported by others^30,31^. On the other hand, the formulations containing both the PS and paclitaxel *i.e*. PSPL, PSPLBC, and PSPLBC + US, exhibited a substantially high cytotoxicity as compared to PSL or Paclitax. It showed that the combination of PS and paclitaxel exerts either additive or synergistic cytotoxic effect on cancer cells. Therefore, to understand the nature of the therapeutic interactions between the PS and paclitaxel, the CI was calculated. The CI for PSPLBC + US was found to be less than 0.1 (synergism) for all the three cell lines. Unlike paclitaxel, the mechanism of PS-induced apoptosis is not clearly understood. Hence, it is difficult to narrow down on the exact mechanism for the observed synergy. Further, the IC_50_ values of the developed formulation for all the three cell lines were calculated and are listed in Table 1.

**Table 1:** IC_50_ (nM) values of different formulations for MDA-MB-231, MCF-7 and B16F10 cells.

Formulation	MDA-MB-231	MCF-7	B16F10
PSPL	14.4 ± 1.3	45.0 ± 1.2	48.2 ± 1.1
PSPLBC	10.0 ± 1.3	49.4 ± 1.3	58.1 ± 1.2
PSPLBC + US	7.6 ± 1.3	36.6 ± 1.2	40.2 ± 1.1
Paclitax	1144.0 ± 1.5	173.2 ± 1.2	240.7 ± 1.2

The IC_50_ values for all the developed formulations were lower as compared to the Paclitax which can be attributed to the observed synergism between PS and paclitaxel. The PSPLBC + US appeared as the most potent of all the formulations with the lowest IC_50_ values for all the cell lines. Based on the previous results, the enhanced cytotoxic potential of the PSPLBC + US can be attributed to the combined effect of, triggered release and enhanced cellular internalization. Further, the substantially low IC_50_ value of the PSPLBC + US compared to Paclitax in a drug resistant cell line (MDA-MB-231), also suggested its potential towards reversal of drug resistance.

### *In vitro* apoptosis assay

These developed formulations contain PS (PSL, PSPL, and PSPLBC) which is a known to induce apoptosis in various cells^30^. Therefore, the apoptotic potential of these formulations was evaluated *in vitro*. The cells treated with these formulations *viz*. PSL, PSPL, PSPLBC, PSPLBC + US, and Paclitax exhibited a significant increase in apoptotic and/or necrotic population (Fig. 4B). This is also supported by the loss of cellular morphology, as observed by the optical microscopy (Figure S8). The PSL treated cells exhibited 34.3% of their population in early apoptotic phase. Since PS is known to play a vital role in apoptotic signaling, it was hypothesized that the PSL treatment leads to exogenous representation of the PS moiety which initiates the apoptotic signal cascade. Hence, the PS-containing formulations were pro-apoptotic. The loading of paclitaxel in these pro-apoptotic vesicles (*i.e*. PSPL), further enhanced their apoptotic potential which was evident from an increase in late apoptotic population (61.4%). This could be attributed to the synergistic action of PS and paclitaxel which in turn accelerated the apoptotic process. Also, contrary to PSPL, the PSPLBC treated cells exhibited only limited apoptotic potential. However, it improved drastically with the application of trigger, and, 11.1% early apoptotic, 73.1% late apoptotic and 7.1% in necrotic population was observed for PSPLBC + US treated cells. These results further confirm that the PS-containing formulations are pro-apoptotic and their apoptotic potential is significantly enhanced with the US-triggered combination therapy.

### *In vitro* serum protein binding, and serum stability

The evaluation of serum protein binding, and serum stability are critical safety parameters for the development of any i.v. formulation. Therefore, the serum protein binding and serum stability of PSPLBC was evaluated at physiological conditions. An adsorption of 23.3 ± 4.7 µg of serum protein per micromoles of PSPLBC lipid was found. This value was consistent with the findings of Johnstone *et al*., using the liposomes of similar lipid composition^32^. The adsorption of serum protein resulted in an increase of the particle size from 780 ± 63 nm to 821 ± 70 nm for PSPLBC and from 206 ± 3 nm to 229 ± 4 nm of PSPL formulations (Fig. 4C). This increase in size is marginal and reflects non-specific serum protein binding. It is desired for reduced clearance by the reticuloendothelial system (RES) and reflects the hemocompatibility of these formulations^32^. Further, no drastic change in the particle size corresponding to agglomeration or degradation was observed. These results indicated the safety, stability, and the hemocompatibility of the PSPLBC.

### Inflammatory response

Non-immunogenicity is a crucial parameter for nanoparticle formulations designed for drug delivery applications. Therefore, the non-immunogenic character of the developed formulations was evaluated. Since, macrophages play a crucial role in host inflammatory immune response to nanoparticle formulations^33^, murine macrophage (RAW 264.7) cells were used for this study. The inflammatory response of the PSPLBC treated macrophages, was estimated from the secreted levels of the pro-inflammatory IL-6, IFN-γ, and TNF-α cytokines. The bacterial lipopolysaccharide (LPS) is a known pro-inflammatory antigen and, hence, was used as a positive control^34^. The media treated cells and TCP served as negative controls in this study^35^. No significant increase in any of the tested cytokines levels as compared to the negative controls were observed for any of formulation treated macrophages (Fig. 4E). This demonstrated the non-immunogenicity of the developed formulations and dismissed the chances of any macrophage-mediated chronic inflammatory response.

## Animal studies

### *In vivo* antitumor efficacy

**Figure 5:**
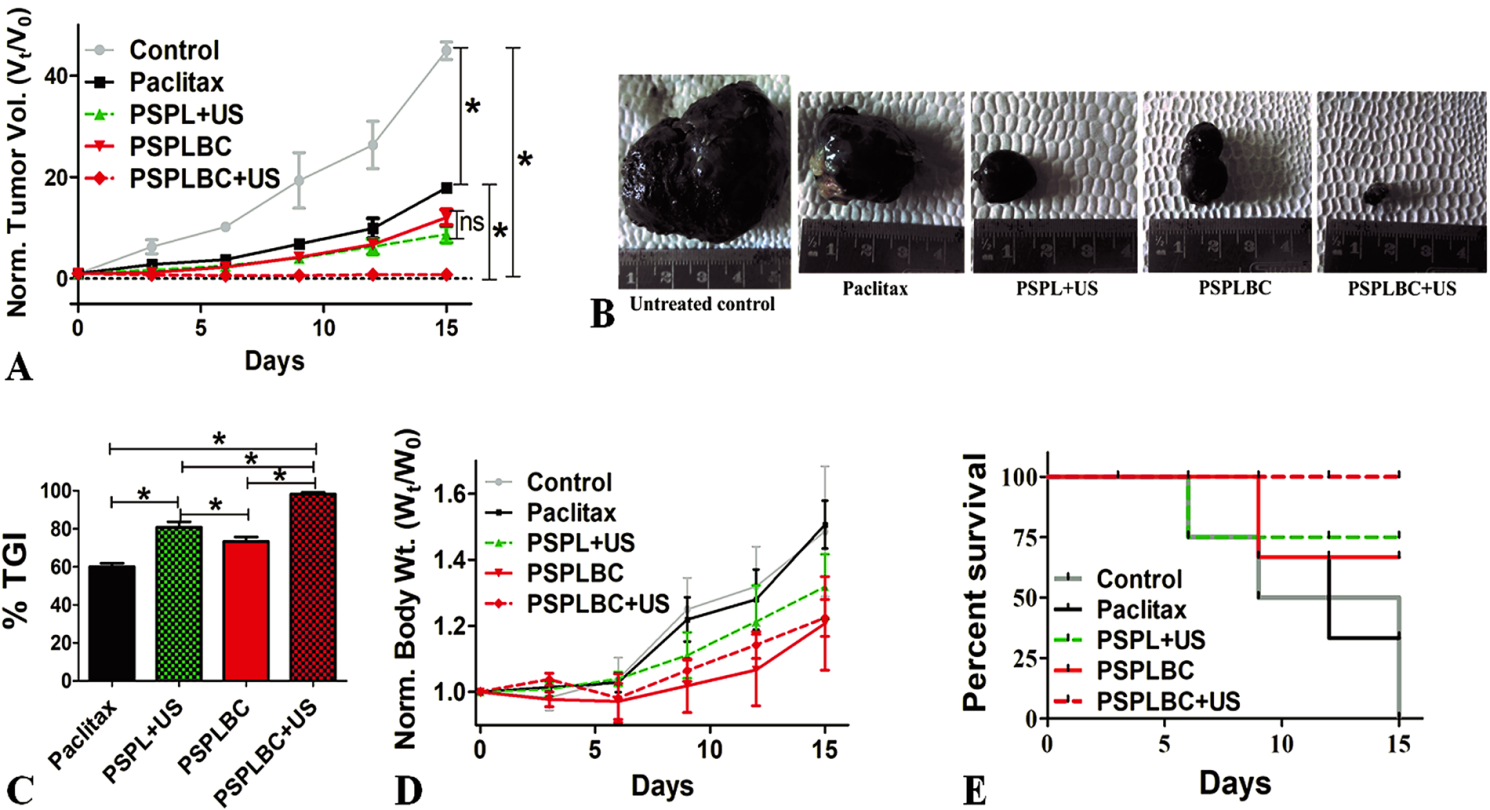
*In vivo* antitumor efficacy and survival study. (**A**) Shows the normalized tumor volume plot for the treated animals. (**B**) Photographs of the excised tumors. (**C**) Shows the percent tumor growth inhibition (%TGI) for the treated groups. (**D**) Shows the normalized body weight of the animals. (**E**) Shows the Kaplan-Meier survival plot (*p ≤ 0.05).

Both melanoma and breast carcinoma are solid tumors and hence, share some commonality. Therefore, the *in vivo* antitumor efficacy was evaluated in murine melanoma model developed in C57BL/6 mice. All the formulations *viz*. Paclitax, PSPL + US, PSPLBC, and PSPLBC + US, exhibited a significant reduction in tumor volume as compared to the placebo control (Fig. 5A). Among the groups, the relative tumor volumes were seen to follow a decreasing trend for Paclitax > PSPLBC ≈ PSPL + US > PSPLBC + US treated group of animals (Fig. 5A). This was also visually displayed by the excised tumor photographs (Fig. 5B). Further, for better representation of the antitumor efficacy, the %TGI was calculated from the relative tumor volumes. The %TGI values were found to be 60.0 ± 1.8% for Paclitax, 80.9 ± 2.8% for PSPL + US, 73.4 ± 2.3% for PSPLBC and 98.3 ± 0.8% for PSPLBC + US (Fig. 5C). It showed that all the developed formulations had better antitumor efficacy as compared to Paclitax which can be attributed to the effective tumor targeting as well as the synergistic action of PS and paclitaxel, as proven *in vitro*. Among the PSPL + US and PSPLBC, the smaller size for PSPL + US proved advantageous for the observed increase in %TGI as compared to PSPLBC. However, with the application of trigger to the PSPLBC administered animals (*i.e*. PSPLBC + US group) the antitumor efficacy was maximum as compared to rest of the groups. These observations could be attributed to the, (a) efficient tumor targeting due to the ability of the PSPLBC to squeeze through neo-vasculature, (b) cavitation-mediated enhancement in cellular permeabilization, and (c) triggered the release of the payload.

Cancer is known to affect the body weight of the organism. Initially, the disease causes weight loss but later the increased tumor burden is reflected in terms of increase in the body weight^14^. Therefore, the body weights of the animals were assessed. The body weight data is in-line with the %TGI data and followed a similar decreasing pattern *i.e*. control > Paclitax > PSPLBC > PSPL + US > PSPLBC + US group (Fig. 5D).

Finally, the study was terminated and animals were sacrificed on day 15 when all the control animals died due to tumor burden. The Kaplan-Meier percent survival rates were calculated to be 100% for PSPLBC + US, 66.7% for PSPLBC, 75% for PSPL + US and 33.3% for Paclitax treated groups (Fig. 5E). The survival data and antitumor efficacy data demonstrated the safety and antitumor efficacy of the developed PSPLBC + US formulation against solid tumors.

### Histological studies

**Figure 6:**
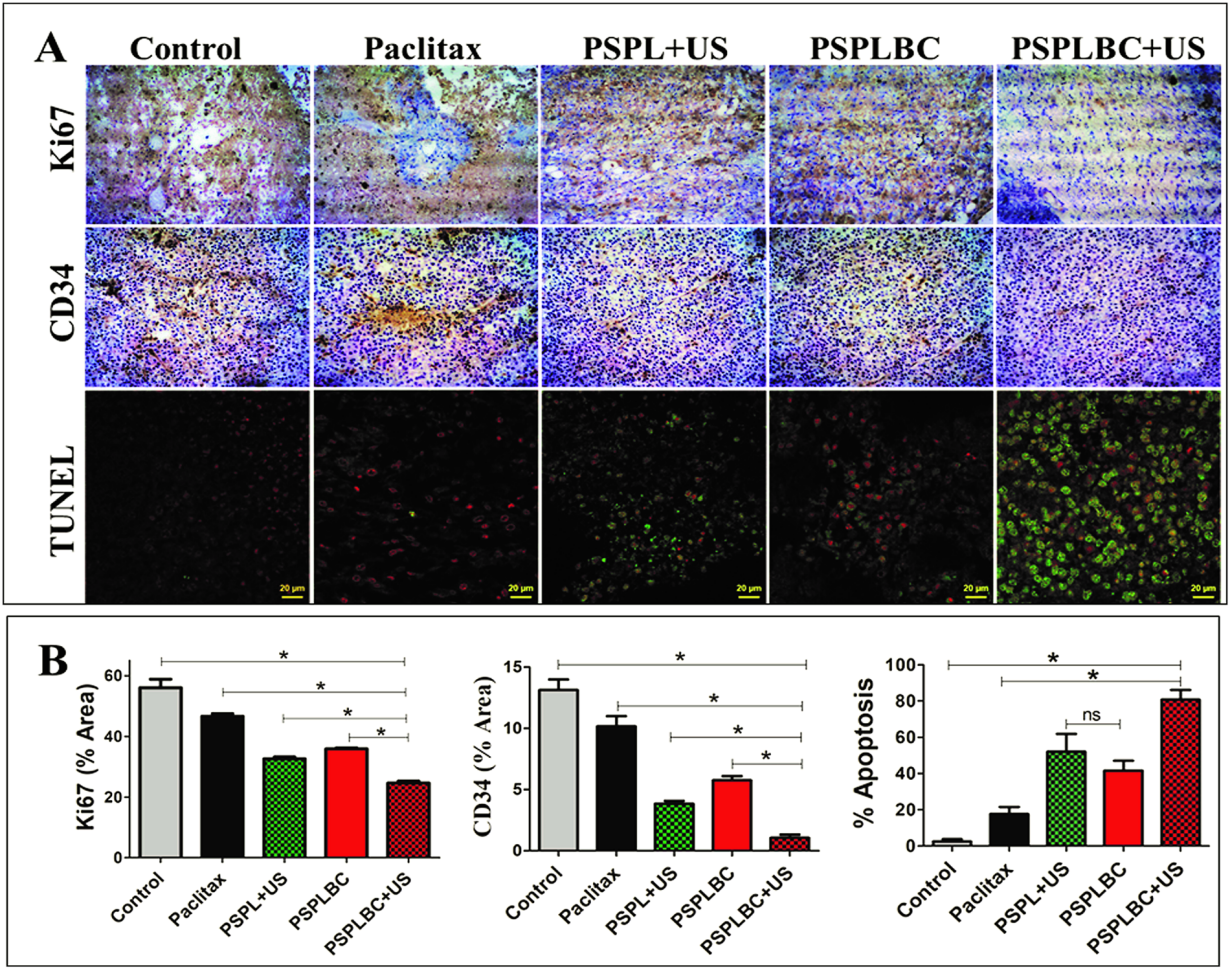
Immunohistochemistry and TUNEL assay. (**A**) Shows the optical images of the Ki67, CD34 and TUNEL stained tumor sections of treated animals groups. (**B**) Shows the percent area covered by the Ki67 positive cells, CD34 positive cells and percent TUNEL positive cells in the tumor sections, (*p ≤ 0.05).

The tumor proliferation index is an important factor in deciding the prognosis of the disease. Usually, higher proliferation index indicates a more virulent disease and vice-versa. Ki67 is a nuclear protein associated with ribosomal RNA transcription and an important cellular proliferation marker^36^. Another major aspect that dictates the prognosis of the disease is the tumor microvessel density (MVD). CD34 is a hematopoietic progenitor cell antigen, a surface glycoprotein. It is expressed on early vascular tissues and hence, an important biomarker for MVD^37^. The reduction in tumor MVD leads to poor blood supply, checking the tumor growth rate. Therefore, the tumor proliferation index and MVD for the treated groups were studied. Figure 6A shows the optical microscopic images of the Ki67 and CD34 stained tumor sections. Both the tumor proliferation index and MVD was found to be in the decreasing order of control > Paclitax > PSPLBC > PSPL + US > PSPLBC + US treated groups (Fig. 6B). Since the tumor proliferation index and the tumor MVD data follows the same pattern as that of antitumor efficacy data, it indicated that the reduction in tumor proliferation index and tumor MVD as the mechanisms for the observed antitumor efficacy. Also, the *in vivo* apoptotic potential of the developed formulations was studied using TUNEL assay. Figure 6A shows the merged confocal images of the TUNEL stained (green) tumor sections with propidium iodide (PI, red) as a counter nuclear stain. The co-localization of green and red signals indicated the TUNEL positive cell (*i.e*. apoptotic cell). The CLSM images clearly showed a substantial increase in co-localized signals for the formulation-treated tumor sections as compared to the control. Further, the TUNEL positive cells were quantified and percent apoptosis was calculated (Fig. 6B). The minimum apoptosis was observed for control group (2.5 ± 1.3%) while, maximum for PSPLBC + US group (80.8 ± 5.3%). For rest of the groups *viz*. Paclitax, PSPL + US, and PSPLBC the percent apoptosis were found to be 17.6 ± 3.9, 52.0 ± 9.9 and 41.6 ± 5.5% respectively. These results confirmed the *in vivo* apoptotic potential of the developed formulations as well as their capability to reduce tumor proliferation index and tumor MVD. These phenomena collectively contribute to the antitumor efficacy of the formulations.

**Figure 7:**
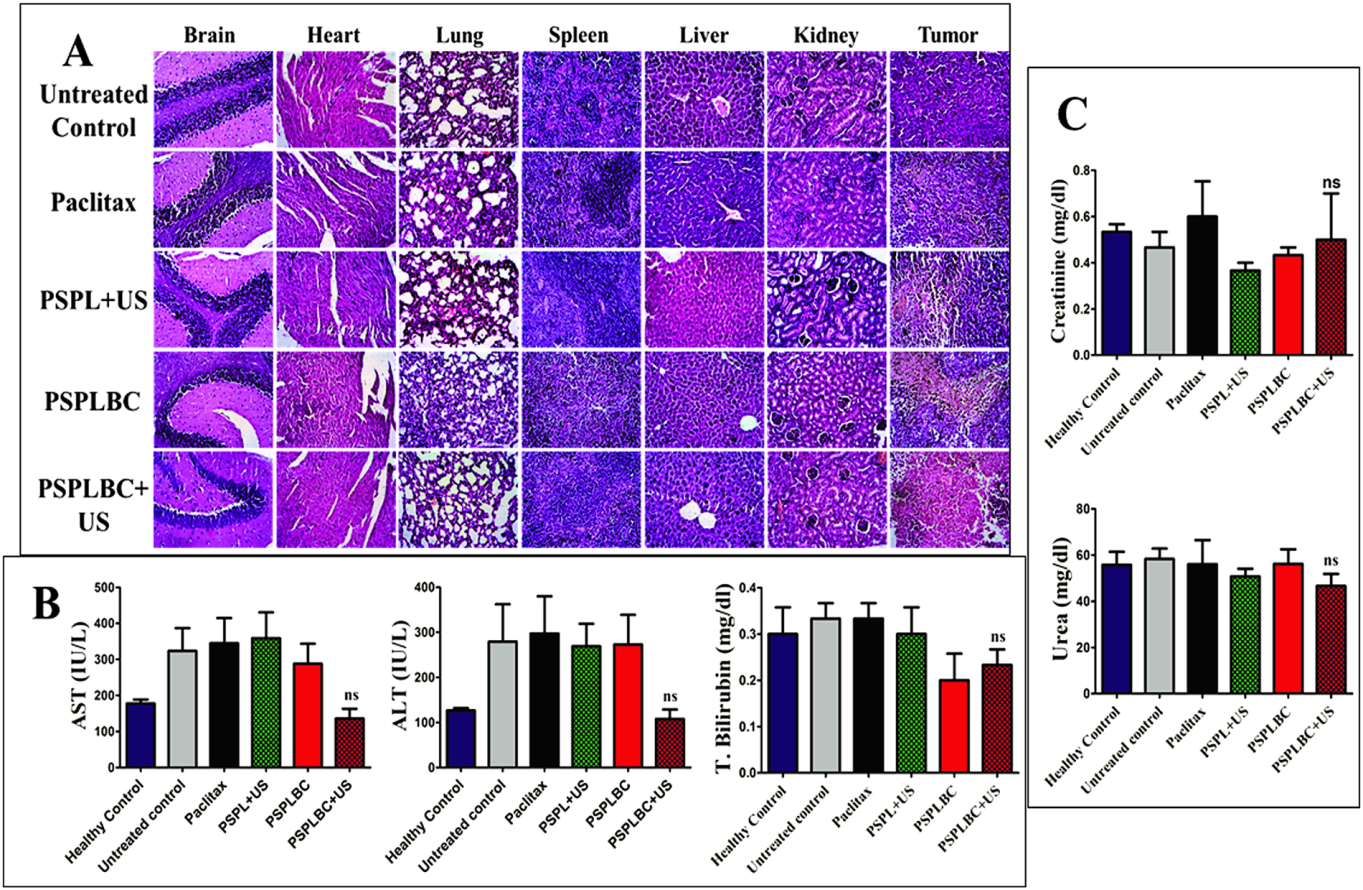
(**A**) H&E staining of the vital organs of the treated animal groups. (**B**) Liver function tests. Aspartate transaminase (AST), alanine transaminase (ALT) and total bilirubin plasma levels. (**C**) Kidney function test. Creatinine and blood urea levels. (ns = not significant).

Next, the histopathology of the vital organs including tumor was studied using H&E staining. No signs of toxicity in terms of necrotic mass or inflammatory infiltrate was observed for any of the vital organs (Fig. 7A). However, necrotic patches were observed in the case of tumor sections of the treatment groups. It shows the efficacy of the formulation in killing the cancer cells *in vivo*. Further, liver and kidney function tests were performed to study the hepato- or nephro-toxicity associated with these formulations. No significant difference in the AST, ALT and total bilirubin levels as compared to healthy control was observed for PSPLBC + US group animals (Fig. 7B). The results indicate the safety of the PSPLBC + US and dismiss any case of severe hepatotoxicity. However, for rest the formulations, the AST and ALT levels were significantly higher but the total bilirubin levels were in normal range as compared to healthy control. It is characterized as mild liver injury, as per the NIH-funded DILI (Drug Induced Liver Injury) guidelines^38^. Since mild hepatotoxicity was observed only for Paclitax, PSPL + US and PSPLBC and no such toxicity were observed for PSPLBC + US. This can be attributed to the systemic hepatotoxicity associated with taxol due to inefficient tumor targeting. Next, the kidney function was accessed by evaluating the creatinine and blood urea levels. No significant difference for both of the functional tests was observed in the case of the formulation treated groups as compared to the healthy control (Fig. 7C). The histopathology data as well as liver and kidney function tests further prove the *in vivo* safety of the PSPLBC + US formulation and dismiss any chances of chronic hepato- or nephro-toxicity.

### *In vivo* ultrasonography

**Figure 8:**
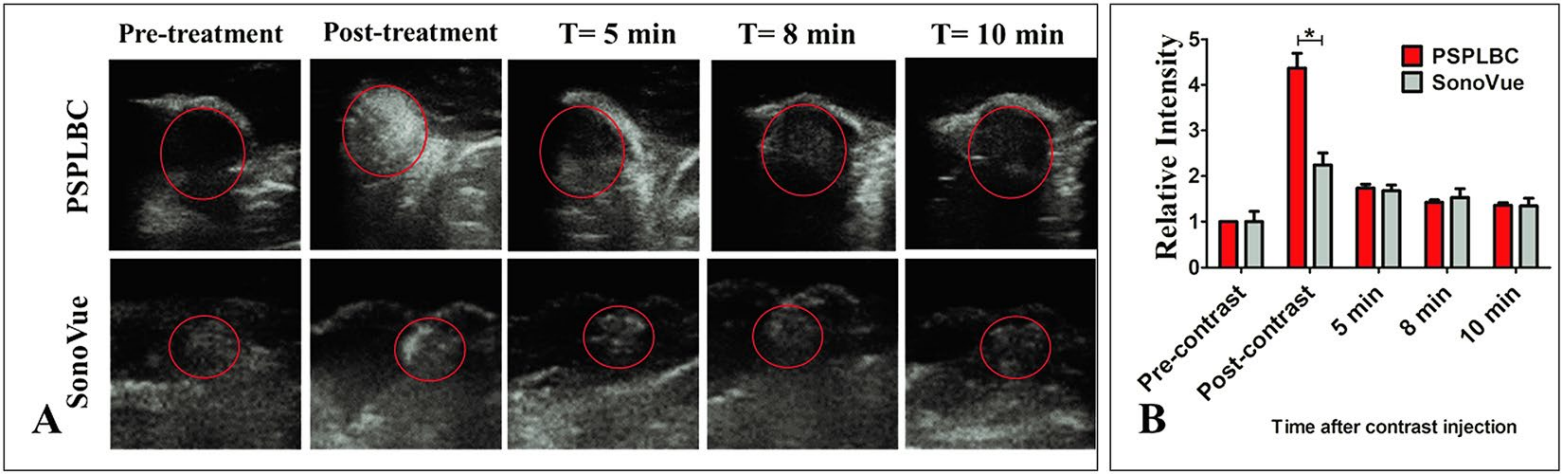
*In vivo* ultrasonography of the implanted tumor in C57BL/6 mice. (**A**) Shows ultrasonographs captured both before and after contrast administration (i.v.). Time-dependent contrast enhancement was followed till 10 minutes of the contrast administration. (**B**) Shows the quantification of the relative time-dependent contrast enhancement by both PSPLBC and SonoVue; (*p  ≤ 0.05).

Finally, the *in vivo* contrast enhancement potential of the PSPLBC was evaluated in tumor-bearing mice model, and compared with that of SonoVue. The standard B-mode sonography was performed at 12 MHz frequency and mechanical index of 0.9 (i.e. 0.66 mW/cm^2^ intensity). The pre-contrast sonography of the tumors exhibited poor contrast which substantially improved immediately after the i.v. administration of contrast agents (Fig. 8A). The contrast enhancement was observed throughout the tumor volume for both the PSPLBC and SonoVue groups. It shows the ability of both the PSPLBC and SonoVue to squeeze through the tumor neovasculature and effectively target the tumor. Further, the tumor gray intensities were quantified and normalized with the native contrast of the respective tumor to obtain the relative tumor contrast (Fig. 8B). It was done to nullify any inhomogeneity arising due to the difference in native contrast of the corresponding tumors. The relative tumor contrast enhancement was found to be 2.2 ± 0.3-fold by SonoVue whereas, 4.4 ± 0.3-fold by PSPLBC; *i.e*. 2-fold increment. This increment can be attributed to the smaller size of the PSPLBC (756 ± 180 nm), giving an edge over SonoVue (2.0–8.0 µm) towards efficient tumor targeting. Both contrast agents showed initial appreciable contrast enhancement which faded with time and only limited contrast was observed over a time of 10 minutes. These results proved enhanced tumor targeting and US-contrast enhancement property of PSPLBC as compared to SonoVue for image-guided therapy of solid tumors. However, this is preliminary proof of concept study for the image-guidance using the standard B-mode (due to technical limitation of the instrument), and requires further evaluation in contrast-enhanced mode.

## Conclusions

To the best of our knowledge, PSPLBC is the only submicron-sized nanobubble-liposome conjugate, composed of a pro-apoptotic biomaterial *i.e*. PS. This study demonstrated the potential of the developed PSPLBC towards image-guided, external trigger-responsive anti-cancer therapy of solid tumors. The findings of this study showed that the PSPLBC was US-responsive and underwent cavitation to exhibit on-demand pulsatile paclitaxel release as well as enhanced the cellular permeability significantly through sonoporation. Also, the incorporation of PS was seen to impart a pro-apoptotic character to the developed formulations which showed synergism with paclitaxel. Combinedly, these features substantially enhanced both the *in vitro* and *in vivo* antitumor efficacy of the PSPLBC + US treated groups. The treated tumor sections exhibited a significant reduction in proliferation index as well as microvessel density (angiogenesis). They also displayed a significant increase in apoptosis. This resulted in significantly high tumor growth inhibition (98.3 ± 0.8%) as well as 100% survival rate of the PSPLBC + US treated animals. Further, the PSPLBC were highly echogenic and enhanced the tumor US-contrast by 4.4 ± 0.3-fold as compared to the native contrast of the tumor and 2-fold as compared to SonoVue. Hence, PSPLBC is a promising, novel, pro-apoptotic, US-responsive drug delivery system for image-guided cancer therapeutics.

## Methods

### Preparation of nanobubbles (B)

SF_6_-gas filled lipid nanobubbles were prepared by sonication method^39^, with minor modifications. Briefly, DSPC:DSPE (4:1, molar ratio) were dissolved in chloroform:methanol (2:1, v/v). The solvents were evaporated using a rotary evaporator to form a thin film, followed by hydration in phosphate buffer saline (PBS) at 60 °C for 45 minutes. Subsequently, the suspension was sonicated (Model 3000 ultrasonic homogenizer, Biologics Inc. USA) with simultaneous gas purging for 30 seconds to form nanobubbles. Similarly, Nile-red dye-loaded nanobubbles were also prepared.

### Preparation of liposomes

PS-liposomes (PSL) were prepared by modified thin film hydration method^40^. Briefly, a thin film of DPPC:DOPS (4:1, molar ratio) was prepared using rotary evaporator and hydrated using PBS (pH 7.4) at 45 °C for 45 minutes. Thus, obtained liposomal suspension (2 mg/ml) was sonicated, and subsequently washed by centrifugation at 25,000 g, 4 °C for 30 minutes. Blank liposomes (BL) containing only DPPC, Paclitaxel-loaded PS-Liposomes (PSPL) containing lipid: drug molar ratio of 2:1 and dye-loaded liposomes (rhodamine-6G or calcein) were prepared similarly. The paclitaxel encapsulation efficiency was calculated by HPLC (Agilent 1100 Binary LC pump liquid chromatography) using C-18 reverse phase column^41^. The encapsulation efficiency was calculated as per equation-1.

1 $$ \% \,Encapsulation\,efficiency=\frac{{A}_{Total}-{A}_{Sup}}{{A}_{Total}}\times 100$$

where A_Total_ is the total amount of paclitaxel used and A_Sup_ is the amount of free paclitaxel in the supernatant.

### Conjugation of nanobubbles and liposomes

Nanobubbles and liposomes were covalently conjugated *via*. amide linkage using EDC/NHS chemistry to form PSPLBC. Briefly, the liposomal suspension was incubated with 0.01% (w/v) of EDC and 0.02% (w/v) of NHS for 15 minutes at room temperature (RT) under gentle stirring to activate the COOH groups. The excess EDC/NHS were removed by centrifugation; followed by mixing of the activated liposomal suspension and nanobubble at RT to form the conjugates. After an hour of incubation in dark, the unconjugated liposomes were removed by centrifugation (5000 rpm, 5 min, 4 °C) and the conjugates (PSPLBC) were resuspended in PBS. Next, the conjugation was optimized using dye-loaded nanobubbles (Nile-red, red) and liposomes (calcein, green) mixed in the ratio of 0.1:9.9, 0.5:9.5, 0.25:9.75 and 1.0:9.0 (v/v) respectively. The percent conjugation was determined by double positive population in fluorescence activated cell sorter (FACS) analysis. Further, these dual dyes loaded conjugates were imaged using a confocal microscope (Zeiss Observer Z1). Also, micron-sized dual dye loaded conjugates were prepared and imaged similarly. Finally, 3D images were created from Z-axis stacks using the Imaris software.

Finally, the PSPLBC suspension containing 10% (w/v) mannitol was lyophilized using Alpha 1–2LDplus (Christ, Germany), and sealed in a glass vial with pressurized SF_6_ gas in the head space (~2 atm). The lyophilized formulation was reconstituted by injecting PBS in the vial through the rubber septum, followed by vigorous shaking for 30 seconds manually.

### Physicochemical characterization

The aqueous colloidal suspensions of BL, PSL, PSPL, B and PSPLBC were characterized for their hydrodynamic diameter and surface charge using dynamic light scattering (DLS) and ZetaPALS (Brookhaven Instruments Corporation, USA) respectively. The stability of the formulation was accessed by monitoring the particle size over time. Next, the particle morphology and size distribution, Transmission Electron Microscopy (TEM, Technai 2, Philips) was studied using the negative staining protocol^42^. Lastly, to study the amide bond formation, Fourier Transformed Infra-Red (FTIR) spectroscopic analysis was done using KBr pellet method (Vertex 80 FTIR System, Bruker, Germany).

### Triggered release

*In vitro* triggered release was studied by dialysis bag method at 37 °C (pH 7.4). Briefly, PSPLBC suspension was packed in pre-soaked dialysis bags and US-trigger (1 MHz, 2 W/cm^2^, 100% Duty Cycle (DC), 60 s, i.e., 11.9 Joules of US-energy per ml of sample) was applied every hour for three hours, using a sonoporator (SP100, Sonidel, Ireland). The dialysis bags were transferred to fresh sink solutions after every sampling. An aliquot was taken at pre-decided time points and paclitaxel was estimated in the sample by HPLC. Further, to study the morphological changes in the structure by the trigger, the PSPLBC samples, both before the trigger and after the trigger were imaged by TEM. The peak negative pressure (PNP) was calculated for the above US-trigger using equation-2.

2 $$\mathrm{Intensity}\,({\rm{I}})=\frac{{({\boldsymbol{PNP}})}^{2}}{{\rm{\rho }}{\bf{c}}}$$

where, ρ = density of the medium, and c = speed of sound in the medium. As, the experiment was conducted in aqueous solution, ρ = 1000 kg/m^3^ and c = 1480 m/s was used for PNP calculation.

### *In vitro* echogenicity

The echogenicity of B and PSPLBC were characterized in contrast enhanced mode using Voluson E8 instrument by GE Healthcare. Briefly, an agarose phantom (1%, w/v) of 12 cm × 5 cm dimension was prepared using degassed distilled water and samples were loaded preformed wells. Ultrasonography was done in both B-mode and CEUS-mode using 2–5 MHz convex phased array probe. The samples were evaluated for their contrast enhancement property and temporal stability under continuous US-exposure, by calculating the pixel intensities of the sonographs using Image J, version 1.47.

### Cell culture

The *in vitro* evaluations were carried out using MDA-MB-231, MCF-7, and B16F10 cells, purchased from National Centre for Cell Sciences (NCCS) Pune, India. Cells were cultured in 25 cm^2^ tissue culture flask (Corning, USA) containing Dulbecco Modified Eagle Medium (DMEM) supplemented with 10% fetal bovine serum (FBS) and 1% antibiotic-antimycotic solution, incubated in a humidified chamber maintained at 37 °C with 5% CO_2_. The spent media was changed every second day with subculture or plating at 70–80% confluency.

### Cellular internalization and mechanism of internalization

The cellular internalization of PSPLBC by MDA-MB-231 and B16F10 cells was studied using confocal laser scanning microscopy (CLSM). Briefly, 10^5^ cells/well was seeded on glass coverslips placed in 24-well tissue culture plates and incubated for 24 hours. The spent medium was replaced by fresh media containing free-dye or rhodamine-loaded PSPLBC and incubated for an hour. US-trigger was applied to the relevant groups. After an hour, the media was removed, cells were washed with PBS and fixed using 4% formaldehyde. The fixed cells were observed under a CLSM (Olympus Fluoview, FV500, Tokyo, Japan) with excitation at 570 nm and emission at 590 nm. The images were acquired with 60 × oil immersion objective using the Fluoview software (Olympus, Tokyo, Japan). The cellular internalization was quantified from the images using Image J, version 1.47. Further, to understand the mechanism of cellular internalization, cells were pre-treated with metabolic and various endocytic inhibitors *viz*. 0.1% sodium azide and 2 μg/ml nystatin^43^, 10 μg/ml phenothiazine^30^, 24 μg/ml cyclosporin-A and 4 μg/ml colchicine for an hour^44–46^. The spent medium was replaced by fresh media containing rhodamine-loaded PSPLBC and further incubated for an hour. Cells were washed, fixed, and imaged, as described earlier. Lastly, the cells were treated with PSPLBC + US and immediately flash-frozen followed by cryo-FEG-SEM imaging (JSM-7600F, Jeol, Tokyo, Japan).

### *In vitro* cytotoxicity study

The dose-dependent cytotoxic potential of BL, PSL, PSPL PSPLBC and Paclitax was evaluated using MDA-MB-231, MCF-7, and B16F10 cells. Briefly, 10^5^ cells/ well were seeded into 24-well plates and incubated for 24 hours. The spent media was replaced with fresh media containing the formulations with equivalent paclitaxel drug concentrations ranging from 10–10,000 nM. US-trigger was applied to the predetermined groups and further incubated for 48 hours. The untreated cells served as negative control. At the end of incubation, MTT assay was performed^47^. The dose-response data points were plotted and IC_50_ values were calculated using GraphPad Prism statistical software. Finally, CI for PS and paclitaxel combination was calculated using Chou and Talalay equation-based CompuSyn, version 1.0 software.

### *In vitro* apoptosis assay

The apoptotic potential of BL, PSL, PSPL, PSPLBC, PSPLBC + US and Paclitax were evaluated using standard annexin-V-FITC and PI dual staining protocol^48,49^. Briefly, cells were seeded in 6-well plate and incubated for 24 hours. The spent media was replaced with fresh media containing formulation (1 µM paclitaxel equivalent) followed by US-exposure to the respective groups. The cells were further incubated for 48 hours. Following the incubation, apoptosis assay was performed according to the manufacturer’s protocol. FACS data was acquired and analyzed using BD FACS Aria (BD Biosciences, USA), and FlowJo software respectively.

### *In vitro* serum protein binding, and serum stability

The *in vitro* serum protein binding, and serum stability were evaluated, as previously reported^50^. PSPLBC suspension was mixed with FBS in varying ratios to obtain a lipid concentration range of 0–1000 µg/ml. As per National Cancer Institute (NCI), USA guidelines, the mixtures were gently mixed and incubated for 2 hours at 37 °C with gentle shaking^51^. The suspension was then centrifuged and the pellet was re-suspended in PBS. To study the serum stability, the particle size was measured using DLS and compared with the untreated sample. For the serum protein binding evaluation, the adhered proteins were extracted as previously reported^52^. The extracted proteins were quantified using BCA protein estimation kit according to the manufacturer’s protocol.

### Inflammatory response

Murine macrophage cells (RAW 264.7) were seeded in 96-well tissue culture plates at a density of 10^4^ cells/well and incubated for 24 hours. The spent media was replaced with fresh media containing PSPLBC at 200 µg/ml lipid concentration and incubated further for 48 hours. The spent media was carefully collected and analyzed for TNF-α, IFN-γ and IL-6 cytokines using ELISA kits (RayBiotech Inc., USA) as per the manufacturer’s protocol. The cells treated with lipopolysaccharide (LPS, 4 µg/ml) served as a positive control while the tissue culture plate (TCP) served as a negative control.

### Animal studies

All the animal studies were approved and conducted as per Institutional Animal Ethical Committee guidelines at National Toxicology Centre (NTC, Pune, India). 6–8 weeks old C57BL/6 mice of 20–25 g weight with mixed male and female population were purchased and used for the studies. Animals were fed *ad libitum* and housed in a constant temperature and humidity conditions with 12-hour light and dark cycle.

### *In vivo* antitumor efficacy

B16F10 murine melanoma cells (1 × 10^6^) suspended in 200 µl saline were subcutaneously injected on the right flank of the animals. The animals were monitored every 2^nd^ day for tumor development. Once a palpable tumor of approximately 100–200 mm^3^ volume was observed, the animals were randomly distributed into five different groups (n = 5) *viz*. control, Paclitax, PSPL + US, PSPLBC, and PSPLBC + US. Day 0 marked the start of the treatment régime, consisted of dosing on every 3^rd^ day (5 doses). All the groups except control received an equivalent dose of 10 mg paclitaxel/kg of body weight as i.v. injection through a tail vein using a 27-gauge needle. US (2 W/cm^2^, 50% DC, 60 s) using sonoporator (SP100, Sonidel, UK) was applied to the respective groups immediately after administration of the formulation. The animal weight and tumor volumes were recorded every 3^rd^ day. The animals were sacrificed on day 15^th^ and organs/tissues were harvested (*viz*. blood, brain, heart, lung, spleen, liver, kidney, and tumor). The Kaplan-Meier survival analysis was done using GraphPad Prism analysis software.

### Histological studies

The tumor section slides were deparaffinized using xylene and stained as per the manufacturer’s protocol for the Ki67 antibody, CD34 antibody, and TUNEL staining. The Ki67 and CD34 stained slides (DAB staining) were visualized using bright field microscopy whereas, the TUNEL stained slides were visualized using CLSM. Three random areas were acquired in each case and analyzed using ImageJ software. For TUNEL positive nuclei, the co-localized green and red (counter stain) signals were counted. Whereas, the total number of nuclei was determined by counting the red signals. Finally, percent apoptosis was calculated as per equation-3.

3 $$ \% \,Apoptosis=\frac{No.of\,TUNEL\,positive\,nuclei}{No.of\,total\,nuceli}\times 100$$

The histopathology involved standard hematoxylin and eosin (H&E) staining. Finally, the liver and kidney function tests were performed from the fresh plasma/serum samples obtained from the blood of the sacrificed animals. For liver function, alanine aminotransferase (ALT), aspartate aminotransferase (AST) and total bilirubin were estimated. While, for kidney function, blood creatinine and urea levels were estimated. All the estimations were done using standard calorimetric methods.

### *In vivo* ultrasonography

The animals were anesthetized using an intramuscular injection of ketamine and hairs in the tumor area was removed using the trimmer. Sonogel was applied on the skin and ultrasonography was performed using 8–12 MHz linear probe (operating at 12 MHz, mechanical index 0.9) attached to MyLab^TM^ Five VET US-scanner (Esaote, India). The intensity of the US-beam was calculated as per the IEC 61102 guidelines^53^. Images were captured in B-mode, both before and after the administration (i.v. through a tail vein) of contrast agents (either PSPLBC or SonoVue). Later the contrast enhancements by PSPLBC and SonoVue were quantified from the respective ultrasonographs (n = 3) using ImageJ software.

### Statistical analysis

All the studies were performed in triplicates and the results were expressed as mean ± standard error. Statistical significance of the data were analyzed by Student’s *t*-test, one-way and two-way ANOVA using GraphPad Prism software. *p* ≤ 0.05 were considered significant for all the analyses.

### Data availability statement

The data and associated protocols will be made promptly available on request without undue qualification for the same.

## Electronic supplementary material

Supplementary Information
