# Supplementary material for: Pro-apoptotic liposomes-nanobubble conjugate synergistic with paclitaxel: a platform for ultrasound responsive image-guided drug delivery
Source: Sci Rep. 2018 Feb 8;8:2624. doi: 10.1038/s41598-018-21084-8 (PMC5805674; doi:10.1038/s41598-018-21084-8)
Supplement: Supplementary file 1 — Supplementary Information [file 41598_2018_21084_MOESM1_ESM.doc]

**Supplementary Information**

**Pro-apoptotic liposomes-nanobubble conjugates synergistic with paclitaxel: a platform for ultrasound responsive image-guided drug delivery**

*Rajeet Chandan^1^ and Rinti Banerjee^1^**

*^1^Department of Biosciences and Bioengineering, Indian Institute of Technology Bombay, Mumbai, Maharashtra, India*

**Materials**

1, 2-dipalmitoyl-sn-glycero-3-phosphocholine (DPPC), 1, 2-distearoyl-sn-glycero-3-phosphocholine (DSPC), 1, 2-distearoyl-sn-glycero-3-phosphoethanolamine (DSPE) and 1, 2-dioleoyl-sn-glycero-3- phospho-L-serine (DOPS) with purity > 99 % were purchased from Avanti Polar Lipids Inc. (Alabaster, AL, USA). SF_6_ gas was purchased from Med Gas & Equipment (Mumbai, India). Dialysis membrane (molecular wt. cut-off 5000-10000), 1-ethyl-3-(3-dimethylaminopropyl) carbodiimide hydrochloride (EDC), N-hydroxysuccinimide (NHS), Dulbecco’s Modified Eagle’s Medium (DMEM), Foetal bovine serum (FBS), Antibiotic & antimycotic solution and Methyl thiazolyl diphenyl tetrazolium bromide (MTT) were purchased from Himedia Laboratories Pvt Ltd. (Mumbai, India). Rhodamine-6G (Rh-6G), Annexin-V-FITC and Hoechst 33342 were purchased from AnaSpec Inc. (San. Jose, CA, USA). Paclitaxel was purchased from RPG life sciences (Mumbai, India). 2-7 Dichlorofluorescein- diacetate (DCFH-DA) and Propidium Iodide (PI) were purchased from Sigma-Aldrich (USA). The recombinant human annexin-V-FITC conjugate was purchased from Invitrogen (CA, USA). Ki67 (ab21700) and CD34 (EP373Y) antibodies were purchased from Abcam. DeadEnd Fluorometric TUNEL System was purchased from Promega. All the reagents and solvents of High-Pressure Liquid Chromatography (HPLC) grade were purchased from Merck. High purity water purified by a Milli-Q Plus water purifier system (Millipore, USA), with a resistivity of 18.2 MΩ-cm, was used in all experiments.

**Buffers:**

- Annexin binding buffer (10mM HEPES, 130mM NaCl, 2.5mM CaCl_2_, pH 7.4)
- Locke's solution (154 mM NaCl, 5.6 mM KC1, 2.3 mM CaCl_2_, 1 mM MgCl_2_, 3.6 mM NaHCO_3_, 15 mM HEPES, 10 mM glucose, pH 7.3)

**EDC/NHS chemistry**


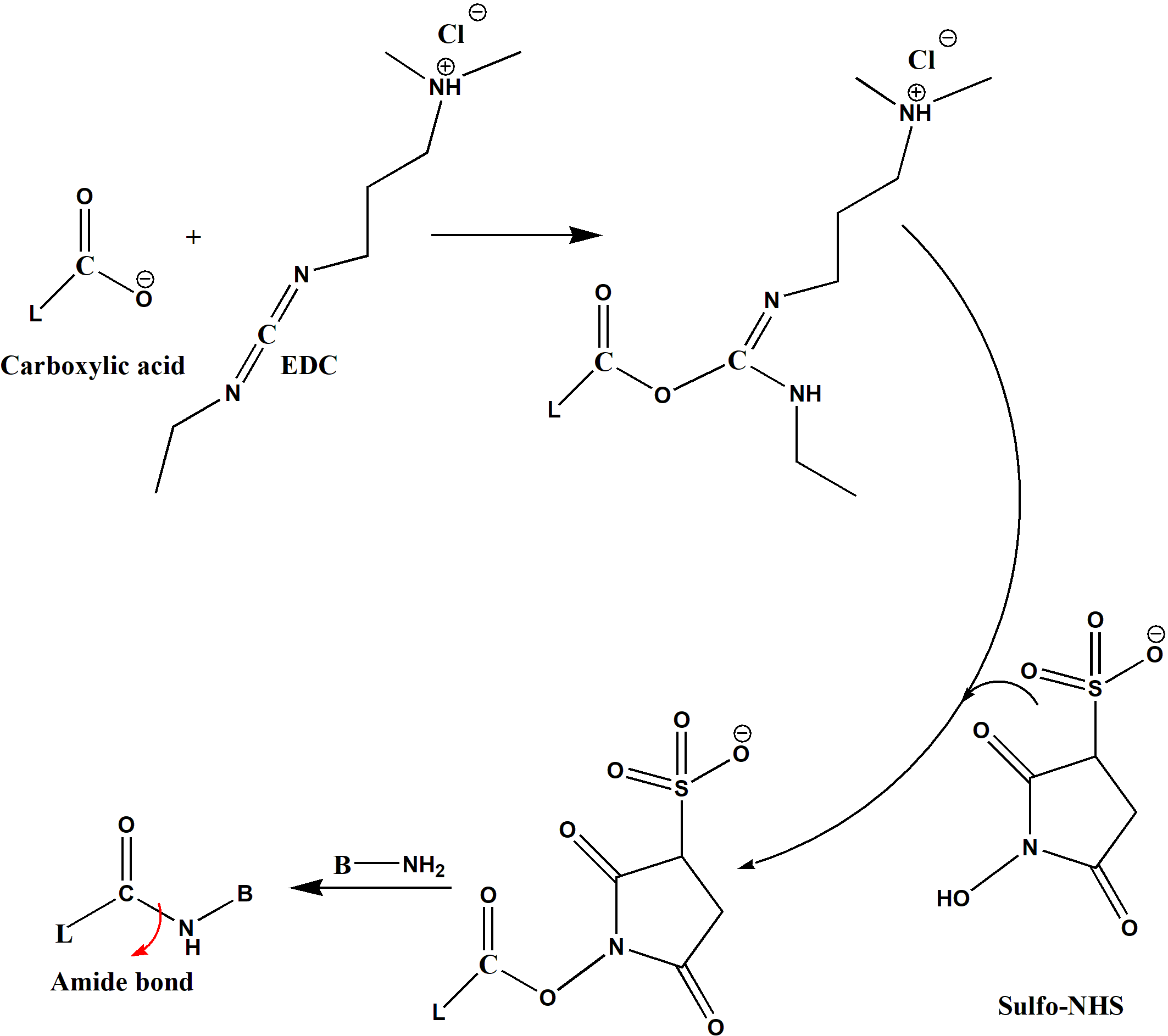


**Figure S1.** Schematic for the standard EDC/NHS amide linkage chemistry; L denotes liposome, while B denotes the nanobubble.

**Optimization of nanobubbles and liposomes conjugation**


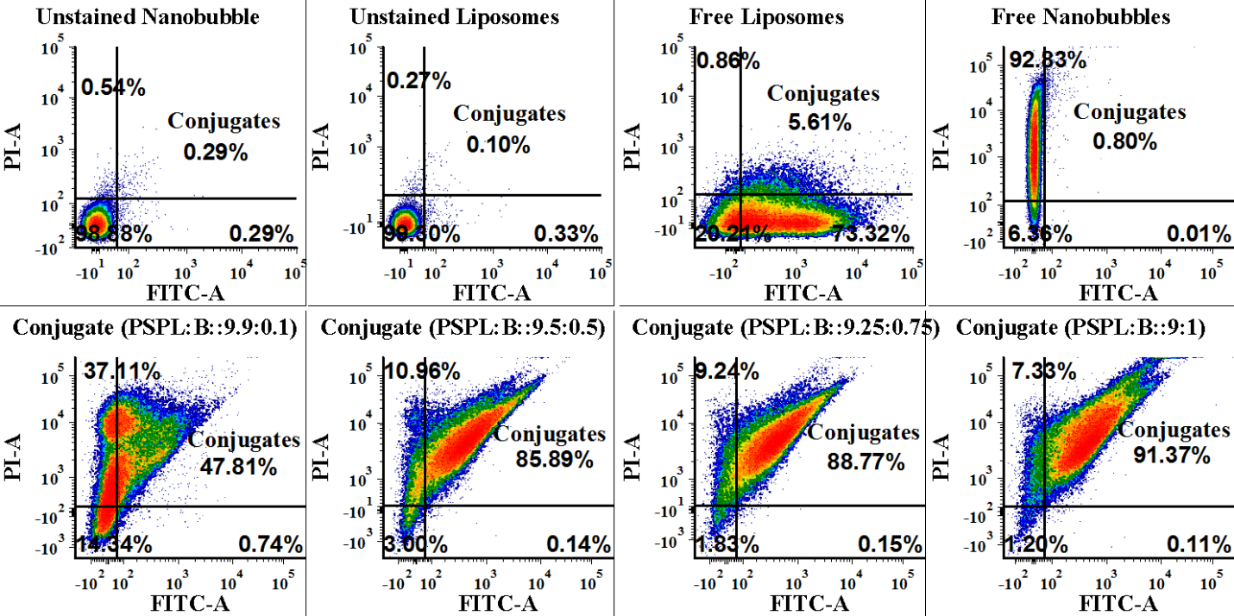


**Figure S2**. Optimization of conjugation efficiency using FACS. The scatter plot of dye-labeled PSPLBC (liposomes – FITC-A channel and nanobubbles – PI-A channel) are shown. The unstained and stained free nanobubbles and liposomes served as controls and the double positive population represented the conjugate population.


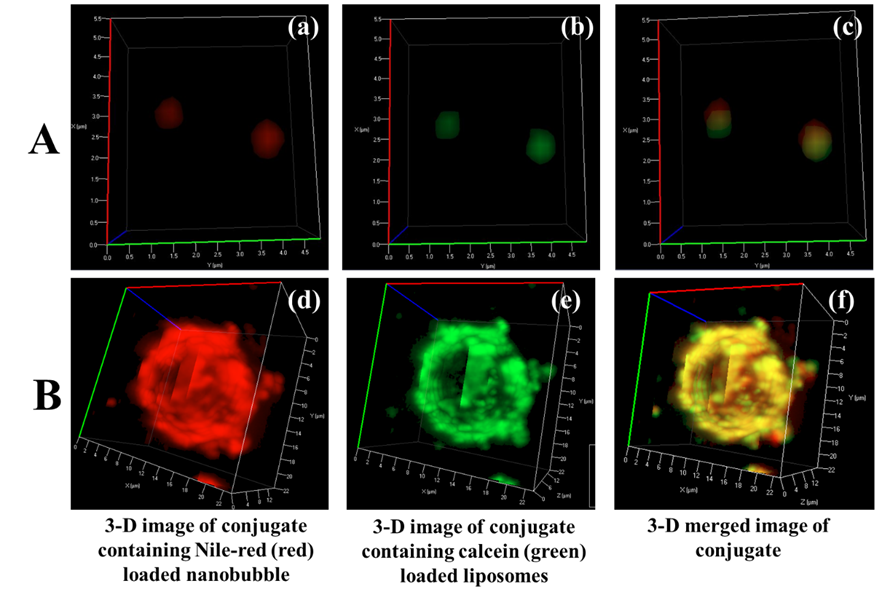


**Figure S3.** Shows the 3-D confocal image of the double dye-labeled PSPLBC. The bubble core was stained with a red dye (NileRed) while liposomes with green dye (calcein). (A) represents the submicron-sized PSPLBC and (B) represents bigger-sized (~16µm) PSPLBC. The merged image shows complete co-localization of two channels on the surface of the sphere suggesting successful conjugation. (Scale bar – panel A, 1 unit = 0.5µm and panel B, 1 unit = 2µm).


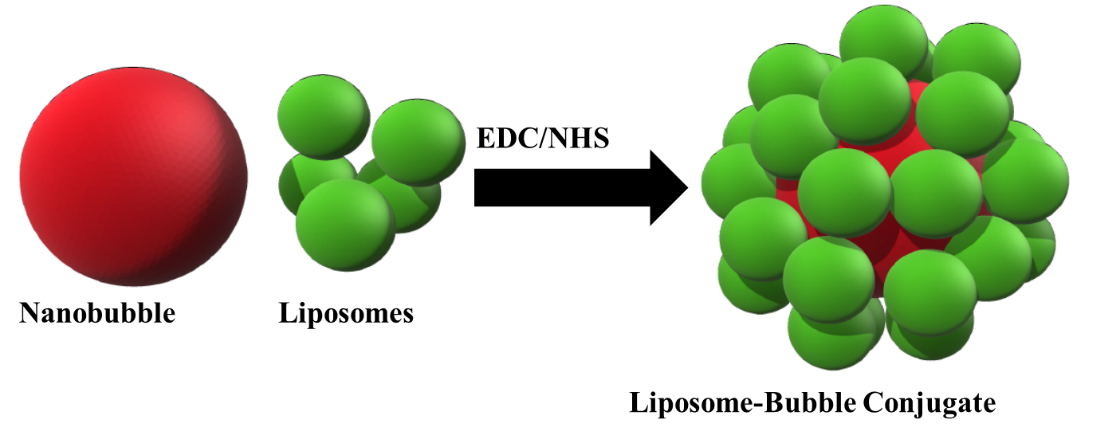


**Figure S4.** Schematic representation of the nanobubble-liposomes conjugation, forming the conjugate (PSPLBC) structure.


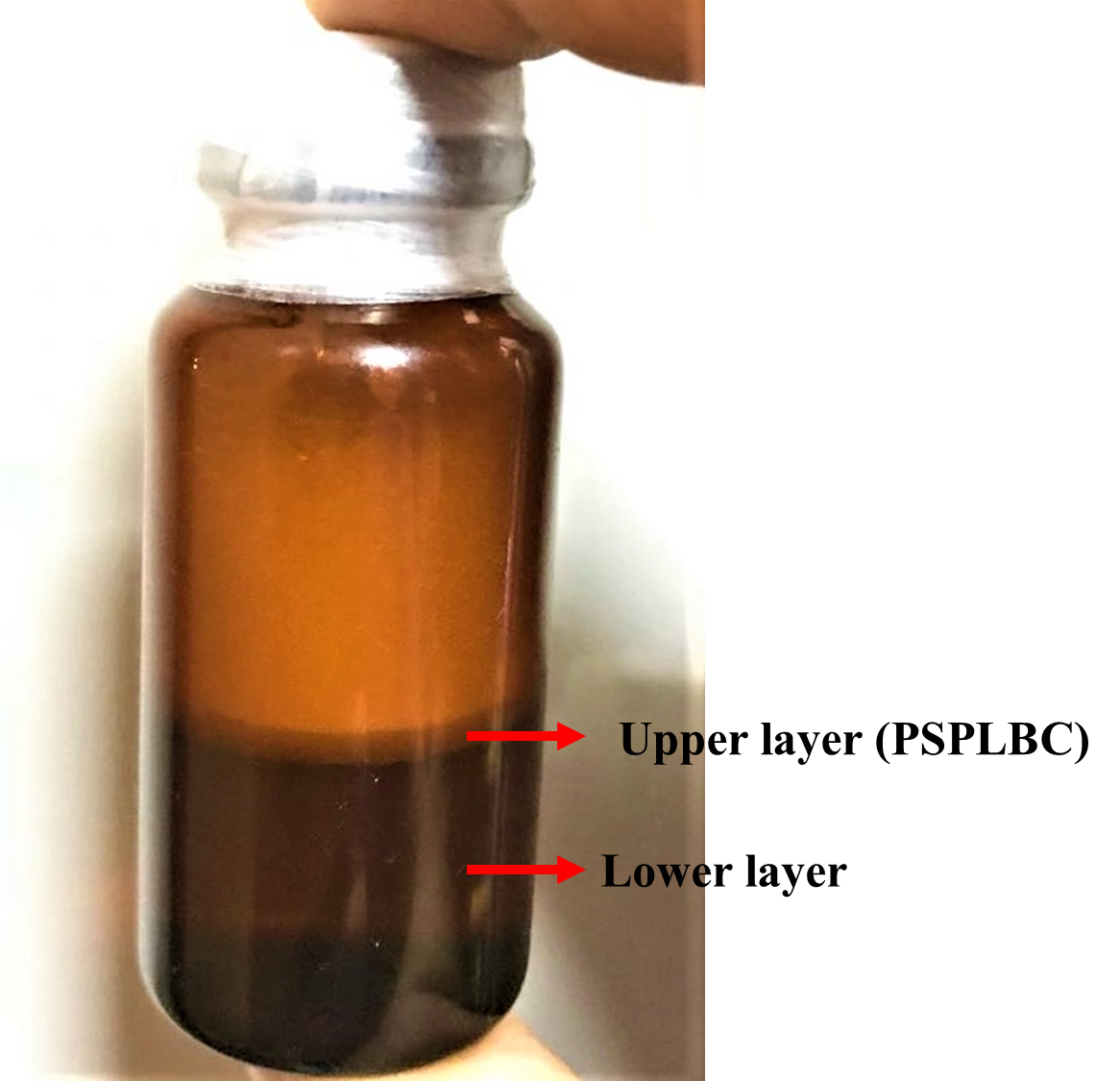


**Figure S5.** The lyophilized PSPLBC formulation reconstituted in PBS with vigorous manual shaking. The suspension separated into lower clear layer and upper thick opaque layer, containing the PSPLBC particles. This gravimetric separation is due to the buoyancy provided by the nanobubble core of the PSPLBC.

**Estimation of residual organic solvents**

Chloroform and methanol were used in the preparatory phase of the formulation. As, these are toxic for the biological samples, and their content should not exceed a threshold in the formulations for biological use. The residual amount of chloroform and methanol was estimated using gas chromatography in the final formulation ^1^. The limit of detection was 100 ppb (0.1 ppm). The chloroform was not detected but trace amount of methanol (1.5 ppm) was estimated in the developed PSPLBC formulation. As per the ICH guideline Q3C (R6) on impurities: guideline for residual solvents, the permissible limit for chloroform is 60 ppm while for methanol is 3000 ppm ^2^. Hence, the residual amount of chloroform and methanol is well under the limits.

***In vitro* US-trigger optimization**

The *in vitro* US-trigger was optimized using MDA-MB-231 cells. Briefly, 1x10^4^ cells/well were seeded in a 24-well plate and incubated for 24 hours. The spent medium was replaced with fresh medium and US-treatment of varying intensity (0-5 w/cm^2^) at 50% duty cycle for 15 seconds was given to cells using sonoporator (SP100, Sonidel, Ireland, UK). The cells were further incubated for 48 hours, followed by MTT (3-(4,5-dimethylthiazol-2-yl)-2,5-diphenyltetrazolium bromide) assay.

Trigger intensity-dependent decrease in percent cell viability was observed (Figure S3). In order to avoid any direct detrimental effect of US on cell viability, more than 95% cell viability was set as the benchmark. Based on the cell viability data, US-trigger of 2 w/cm2, 50% duty cycle for 15 seconds (2/50/15) was selected as US trigger for all the *in vitro* studies.


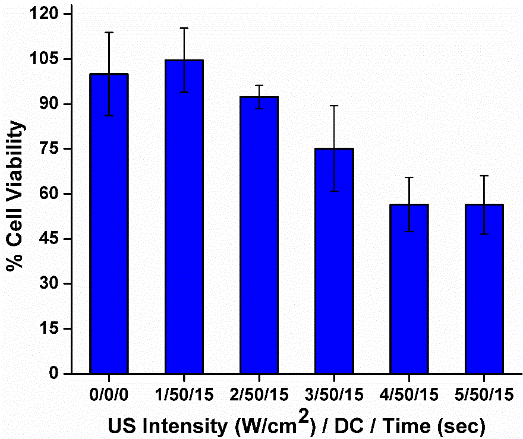


**Figure S6.** In vitro US trigger optimization study. Cell viability of MDA-MB-231 cells exposed to US trigger of varying intensity (0-5 w/cm2) with fixed 50% duty cycle (DC) and 15 seconds, as estimated by MTT assay.

The cell viability of the MDA-MB-231 cells treated with PSPLBC (10 nM) + US -trigger or US-trigger alone was also evaluated by MTT assay, as described in the manuscript. The US-trigger of 1 MHz, 2 W/cm^2^, 100% DC, 60 s (same as drug release study), was used for this study. The cell viability was found to be 3.5±2.5% for the PSPLBC+US-trigger as compared to 5.6±3.8% for cells treated with US alone (no significant difference); i.e. US-alone is killing the cells. This data supports the cell viability versus US-intensity trend observed in figure S6. The US-energy applied in this experiment is 96 Joules/ml as compared to the optimized value of 12 Joules/ml, used for all the *in vitro* cellular studies. It shows that the US-energy per unit sample, above a threshold is detrimental to the cells.


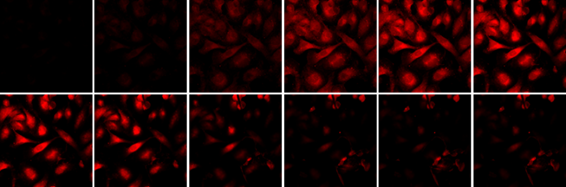


**Figure S7.** Z-stack confocal images of the cells treated with the dye-loaded PSPLBC. The images were captured in Z-range of -10µm to +10 µm at an interval of 1 µm using 63x oil immersion objective lens.


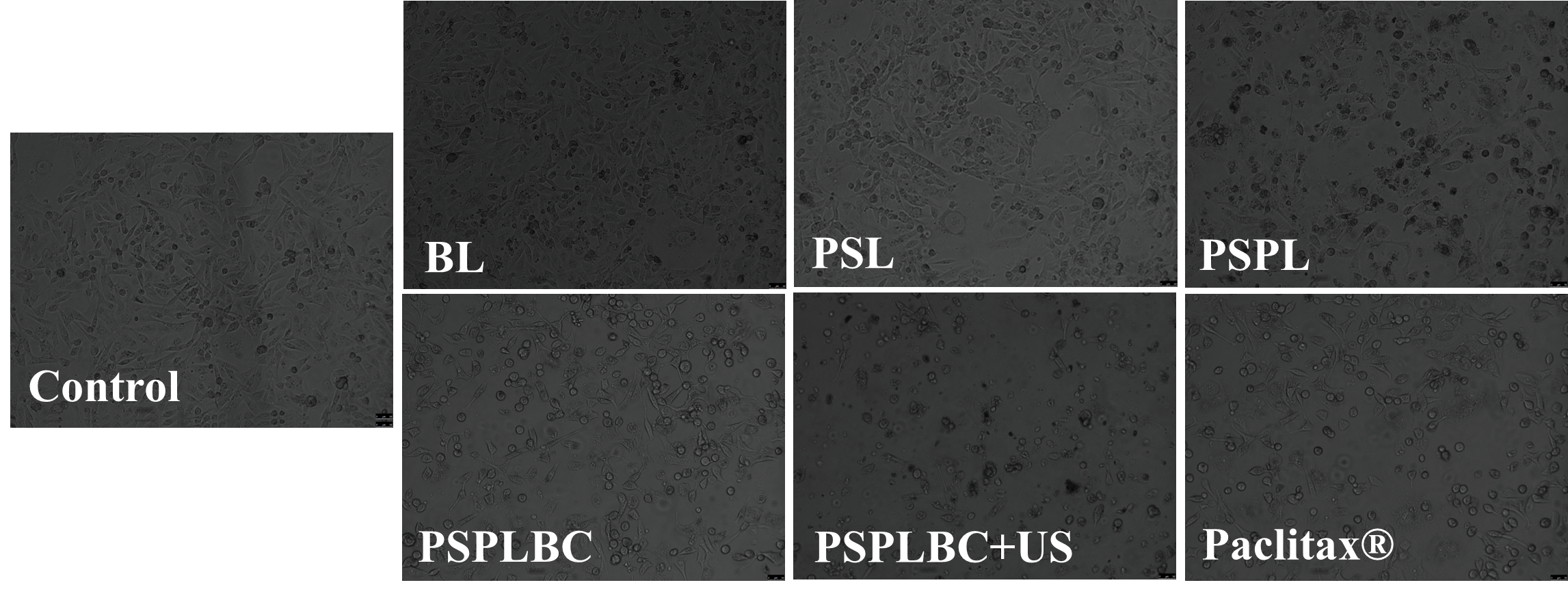


**Figure S8.** Optical microscopic images of the cells treated with BL, PSL, PSPL, PSPLBC, PSPLBC+US, and Paclitax® showing the alteration in cellular morphology due to apoptosis. The images were captured at 10X to include a maximum number of cells per frame.

**References**

1. Puranik, S. B., Pai, R., Pai, P. N. S. & Rao, G. K. Gas chromatographic determination of residual levels of methanol and chloroform from liposomal, microspheres and nanoparticles. **6,** 693–704 (2008).

2. European Medicines Agency. ICH guideline Q3C ( R6 ) on impurities : guideline for residual solvents. *Eur. Med. Agency* **44,** (2017).
